# Direct Detection of the Biological Toxin in Acidic Environment by Electrochemical Impedimetric Immunosensor

**DOI:** 10.3390/s101211414

**Published:** 2010-12-13

**Authors:** Changhoon Chai, Jooyoung Lee, Paul Takhistov

**Affiliations:** School of Environmental and Biological Sciences, Rutgers, the State University of New Jersey, New Brunswick, NJ 08901, USA; E-Mails: changhoonchai@gmail.com (C.C.); jooyoung@alumni.rutgers.edu (J.L.)

**Keywords:** hydrophobic surface modification, immunosensor, direct detection, electrochemical impedance, nano-porous aluminum, antibody immobilization, biological toxin, ricin, acidic foods

## Abstract

This study describes the direct detection of the biological toxin (Ricin) in acidic environment without pH adjustment by hydrophobically modified electrochemical impedance immunosensor (EII). The nano-porous aluminum substrate for EII was hydrophobically modified via self-assembled monolayer (SAM) of APTES. Biosensor for the detection of the Ricin was fabricated by the covalent cross-linking of antibody (Ab) with APTES-SAM. The immunoreactions between the immobilized Ab and the biological toxin in several diagnostic solutions were monitored by the electrochemical impedance spectroscopy (EIS) under the polarization of EII *versus* reference electrode. EII could detect the presence of the biological toxin in acidic foods in 20 mins without pH adjustment. The negatively charged ions including hydroxides would be adsorbed on the hydrophobic body of APTES-SAMs by the polarization during EIS analysis, and offset the effect of acids on the immunological activity of the immobilized Ab. It suggested that the adsorption of negatively charged ions helped to keep the immunological activities of the immobilized Ab on EII in acidic environment. Proposed mechanism of the localized pH adjustment that makes possible immunoreaction occurrence in low pH sample matrix is briefly discussed.

## 1. Introduction

Detection of biological toxins is on the practical front line to keep public health and food safety from foodborne illness and bioterrorism [1]. As the efficient protection is of concern to public health and food safety, the detection method has to be sensitive, rapid, and applicable to diverse sorts of diagnostic samples. However, it is the challenge to develop the immunosensor for the direct detection of biological toxins in the acidic diagnostic samples since acids interfere with immunoreaction [2]. Although the acidic environment is regarded as microbiologically safe, the acidic environment is the proper condition where some of biological toxins firmly maintain their configurations and toxicities. Additionally there still exist the potentials of foodborne illness from acidic foods, hence Food and Drug Administration of United States (FDA) regulates acidic foods by classifying into low acid foods and acid foods [3,4]. Generally immunoreaction occurs around neutral pH, and the rate of immunoreaction is extremely low in acidic environment. Accordingly traditional immunosensors such as ELISA (enzyme-linked immunosorbent assay), RIA (radioimmunoassay), and electrophoretic immunoassay frequently include the step of pH adjustment prior to the analysis of target toxins in order to avoid the interference by acids on immunoreaction.

In the immunosensing system, the detection of biological toxin is accomplished through the continuous sequence of physicochemical processes: (1) the diffusion of the biological toxin from the solution of a diagnostic sample to the solid-liquid interface between immunosensor and the diagnostic solution, (2) the immunoreaction between the toxin and the molecular recognition elements, *i.e.*, antibody (Ab), antigen (Ag) ligand, and *etc.*, and (3) the transduction of the immunoreaction to analytical signals in the immunosensing system. The diffusion of biological toxin from the solution to a solid-liquid interface is mediated by solvent. Ions dissolved in the diagnostic sample are diffused to the solid-liquid interface along with biological toxin. Diffused ions including acids would affect the immunoreaction of the molecular recognition elements.

The behavior of ions at the solid-liquid interface shows the different tendency opposed to the surface properties. Ions are absorbed on the surface that has high dielectric constant. They also would be repelled from the surface having low dielectric constant, such as hydrophobic surface [5]. Hydroxide, however, interestingly tends to adsorb at the water-hydrophobic interface [5–7]. As a consequence of different behaviors of acids and hydroxides on the hydrophobic surface, we assumed that the effect of acids on the immunological actions of molecular recognition elements would be offset or reduced by the hydrophobic modification of immunosensor’s surface.

In this study, ricin and anti-ricin were selected to demonstrate the Ag-Ab reaction as a model immunosensing system since ricin, a lethal biological toxin, which has been used as a biological warfare, keeps its stability and toxicity in acidic environment. Aluminum substrates were hydrophobically modified with 3-aminopropyltriethoxysilane (APTES), then the immunosensor for the detection of ricin was prepared by the covalent immobilization of anti-ricin on APTES treated aluminum substrate. Immunoreaction between Ab on immunosensor and Ag in acidic environment was monitored based on electrochemical impedance spectroscopy (EIS) since electrochemical impedimetric immunosensor (EII) has been shown an advantage to directly detect broad organic substances including DNA [8], proteins [9], microbes [10], and biological toxin [11] without labeling. The accessibility of ions onto Ag immunosensor was analyzed and the immunological activity of immobilized Ab in acidic environment was investigated. Then the application of developed EII to acidic foods was demonstrated.

## 2. Experimental Section

### 2.1. Materials

Oxalic acid (anhydrous 98%) and phosphoric acid (85% water solution) were purchased from Acros Organics (NJ, USA). Ethanol and acetone were obtained from Fisher Scientific (Fair Lawn, NJ, USA). Perchloric acid (60%) and 2-butoxyethanol were obtained from Alfa Aesar (Ward Hill, MA, USA) and J.T. Baker (Phillipsburg, NJ, USA), respectively. 98% of 3-aminopropyltriethoxysilane (APTES) was purchased from Strem Chemicals (Newburyport, MA, USA). Glutaraldehyde (70% v/v, Grade I), ethanolamine (98%), and sodium chloride (99%) were purchased from Sigma Aldrich (St. Louis, MO, USA). Electropolishing solution was prepared by mixing 70.0 vol.% of ethanol, 13.8 vol.% of distilled water, 10.0 vol.% of 2-butoxyethanol, and 6.2 vol.% of perchloric acid. Both polyclonal anti-ricin (Ab) and ricin (Ag) were purchased from Toxin technology Inc. (Sarasota, FL, USA). Commercial food-grade aluminum (alloy 1100, thickness 0.25 mm), PTFE, polycarbonate, and stainless steel 316 were purchased from McMaster-Carr (Dayton, NJ, USA). The custom-designed electrochemical chamber was developed for electropolishing, anodization, and EIS analysis. Its body was made of polycarbonate. It consists of a disk-shaped stainless steel counter electrode (CE, diameter 30.5 mm), steel support for the working electrode (WE, diameter 12.8 mm), and a placeholder for the reference electrode (RE). The internal compartment of the chamber is conically-shaped. This allows to use the counter electrode with larger surface area than that of a working electrode (*S_CE_*/*S_WE_* *∼* 10), which minimizes the effects of CE polarization on the sensor’s signal.

### 2.2. Methods

#### Preparation of nano-porous substrates for immunosensor

The process of nano-patterning was carried out on each substrate followed by three steps: annealing at 500 °C, electropolishing, and anodization. Such sequential process created reproducible nano-patterns on aluminum substrates. Alloy 1100 was cut into 12.8 mm in diameter and anealed at 500 °C after cleaning the surface with acetone. To get smooth surface, the aluminum substrates were polished electrochemically in the electropolishing solution for 40 sec with vigorous stirring at 42 V by PC-controlled DC power supply (1787A, BK Precision Corp., Yorba Linda, CA, USA) [12]. After the electropolishing step of the substrate, the aluminum anodization was performed in 0.3 M oxalic acid at 40 V. During the anodization, the temperature was maintained at 5 °C by refrigerated circulator (3016, Fisher Scientific, Pittsburgh, PA, USA). Prepared nano-porous aluminum substrates were then washed with SDI water and dried in nitrogen atmosphere. Finally, nano-porous aluminum substrates were heated at 150 °C and stored in a sterilized chamber to prevent the accidental contamination.

#### Sensibilization of nano-porous aluminum

Electrochemically processed nano-porous aluminum substrates were silanized in 2 vol.% APTES for 4 hrs. Silanized aluminum surface was activated in 2.5 vol.% glutaraldehyde for 2 hrs. Activated aluminum disc was placed in the solution Scontaining 40 μg/mL Ab in 0.01 M phosphate buffer (pH 7.0) at 37 °C for 1 hr and, further, at 4 °C for 12 hrs. To prevent non-specific binding of alien substances on the surface of EII as well as to improve the specificity of EII to the target Ag, remaining prospectively vacant sites on the surface were blocked by soaking in 100 mM ethanolamine solution for 1 hr [13]. Prepared EII was thoroughly cleaned out with SDI water and dried in nitrogen. Then fabricated sensors were stored in sterilized containers at −25 °C up to 3 months.

#### Surface morphology analysis

The surface morphology of EII was observed by SPM Q-Scope 350 (Quesant Inst. Corp., Agoura Hills, CA, USA) in tapping mode with NSC-16 cantilevers. The specialized software package, SPIP 3.3 (NanoScience, Phoenix, AZ, USA) was used for image processing.

#### Electrochemical analysis

Impedimetric and cyclic voltammetric responses of EII were analyzed by PC-controlled electrochemical workstation DHC2, equipped with PC4 (750)/DC105 potentiostat (Gamry Instruments, Warminster, PA, USA). Double junction Ag/AgCl reference electrode PHE 3211 (Omega Engineering Inc, Stamford, CT, USA) was used for electrochemical impedance spectroscopy (EIS) and cyclic voltammetry (CV). 0.3% NaCl solution that was used for the preparation of Ag solutions was conditioned by purging high-pure nitrogen prior to the electrochemical measurements. EIS and CV of EII in the differently concentrated Ag solutions (0, 0.1, 0.5, 1, and 10 μg/mL in 0.3% NaCl) were obtained at predefined time intervals. Electrochemical impedance at single frequency (10 kHz) was carried out with triplicate to investigate the reproducibility of immunosensing ability of developed EII in this study, and obtained impedimetric parameter was presented as average with standard deviation. EIS was carried out in the frequency range from 0.25 Hz to 100 kHz with the excitation voltage of 10 mV. For EIS analysis, EII was polarized with Δ*ϕ* = +0.1 V *vs*. Ag/AgCl electrode. CV was performed in the voltage range of −2.0 + 1.0 V *vs*. Ag/AgCl reference electrode at the scan rate of 100 mV/s.

## 3. Results and Discussion

### 3.1. Hydrophobic Surface Modification and EII Preparation

**Figure 1.**
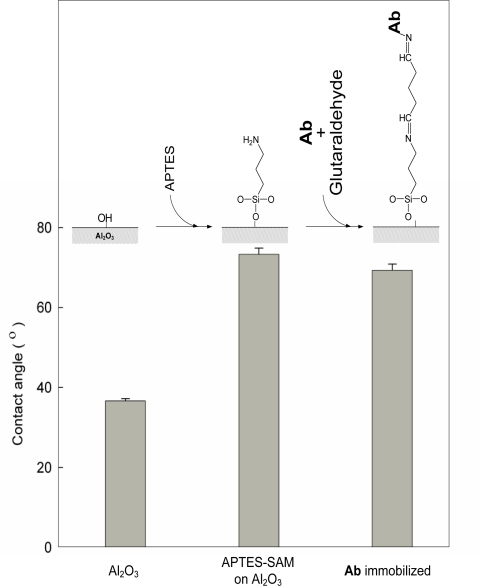
The schematic design of the deposition of APTES-SAMs and Ab immobilization on aluminum surface, and the changes of water contact angles on aluminum substrates as the formation of APTES-SAMs and Ab immobilization.

Aluminum is covered with naturally enhanced aluminum oxide layer, which is chemically and electrochemically reactive. Therefore, the aluminum surface can be chemically modified by the deposition of chemicals such as siloxane agents, and fabricated to nano-porous structure by the electrochemical processes such as anodization. In this study, the aluminum substrate was silanized with APTES to modify its surface property to hydrophobic (Figure 1). Self assembled monolayers (SAMs) of APTES (APTES-SAMs) were deposited on the surface of the aluminum substrate by siloxane linkage with hydroxyl groups on aluminum oxide layer (Figure 1). Then Ab was covalently immobilized on APTES-SAMs by the addition of a linker, glutaraldehyde.

In order to avoid the geometric effect on the contact angle, the measurement of water contact angle was executed on EII based on planar aluminum substrate. Due to the hydrophobic moiety of an aliphatic chain of APTES, the deposition of APTES-SAMs on the aluminum surface changed the surface property to hydrophobic, which was observed by the increased water contact angle (Figure 1). Despite of Ab immobilization, it didn’t make the significant change of the surface property as for the hydrophobicity of surface. It was expected that the hydrophobicity arising from APTES-SAMs after Ab immobilization would help to maintain the immunological activity of immobilized Ab in acidic environment by adsorbing hydroxides near hydrophobic APTES-SAMs.

**Figure 2.**
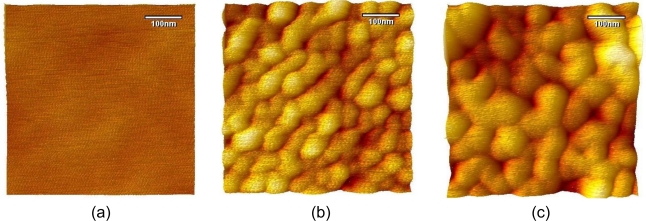
AFM images of **(a)** plain aluminum substrate, **(b)** nano-porous aluminum substrate, and **(c)** EII.

However, for the purpose of the good sensitivity, EII used for the investigation on electrochemical response of EII to immunoreaction was developed based on nano-porous aluminum substrate [11]. EII was prepared on nano-porous aluminum substrate by the deposition of APTES-SAMs with a subsequent Ab immobilization. Aluminum oxide layer on aluminum substrate grew to a well-ordered nano-porous structure by the anodization in 0.3% oxalic acid at 40 V (Figure 2(b)). The surface morphology analysis of EII shows the intimate and structured incorporation of immobilized Ab with nano-porous aluminum substrate (Figure 2(c)).

### 3.2. Electrochemical Characterization of EII

Impedimetric response of EII to the immunoreaction between immobilized Ab on EII and Ag in a diagnostic solution was characterized based on EIS technique [11]. From EIS analysis, we obtained the complex impedance (*Z*), which can be represented as:

(1) $$Z(t,\;\omega )=\frac{V(t,\;\omega )}{I(t,\;\omega )}=Z_{0}(t)e^{j\theta }=Z^{\prime }(t,\;\omega )+Z^{\prime \prime }(t,\;\omega )$$

where *Z*(*t*, *ω*) is a complex impedance (*Z*) at time (*t*) and radial frequency (*ω*), *V* is a voltage, *I* is a current, *θ* is a phase angle, *Z’* is the real part of a complex impedance, and *Z”* is the imaginary part of the complex impedance. The changes in the physical and/or chemical properties at the solid-liquid interface between EII and the diagnostic solution by the immunoreaction reproducibly responded specifically to *Z’* under the polarization of EII *versus* reference electrode. As *Z’* is the impedimetric parameter representing inductive characteristics, the interfacial changes of EII by the immunoreaction would alter inductive characteristics. The immunoreaction on the surface of EII was monitored by the analysis of *Z’*. Obtained *Z’* from EIS analysis of EII was normalized to compare *Z’* from EII in various experimental conditions:

(2) $$Z_{\text{norm}}^{\prime }(t,\;\omega )=\frac{Z^{\prime }(t,\;\omega )}{Z^{\prime }(0,\;\omega )}$$

where *Z’_norm_*(*t*, *ω*) is a normalized *Z’* at *t* and *ω*.

**Figure 3.**
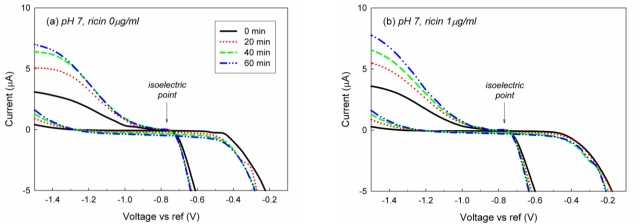
Changes on the cyclic voltammograms of EII at pH 7 in **(a)** Ag 0 μg/mL and **(b)** Ag 1 μg/mL. Ricin, the biological toxin, was used as Ag of EII.

It has been reported that EII detects target toxin faster and more sensitive when impedance is analyzed under the polarization of EII *versus* reference electrode [11]. In this study, we also adopted the polarization method of EIS to achieve a good sensitivity and speed of EII. The polarization of EII at +0.1 V *versus* reference electrode during EIS analysis would attract negatively charged ions including hydroxides to the surface of EII. The accessibility of the ions to the surface of EII under the polarization could be interpreted by the analysis of isoelectric point (IEP) of EII based on cyclic voltammetry (CV). Figure presents cyclic voltammograms (CVs) of EII in pH 7, Ag 0 μg/mL and pH 7, Ag 1 μg/mL. IEP of EII is around −0.765 V regardless of the presence of Ag (Figure 3). IEP of EII is corresponding to IEP of bare aluminum [14]. Accordingly under the polarization, negatively charged ions would reach to the aluminum surface of EII passing through Ab layers and APTES-SAMs.

### 3.3. Immunological Activity of Immobilized Ab in Acidic Environment

**Figure 4.**
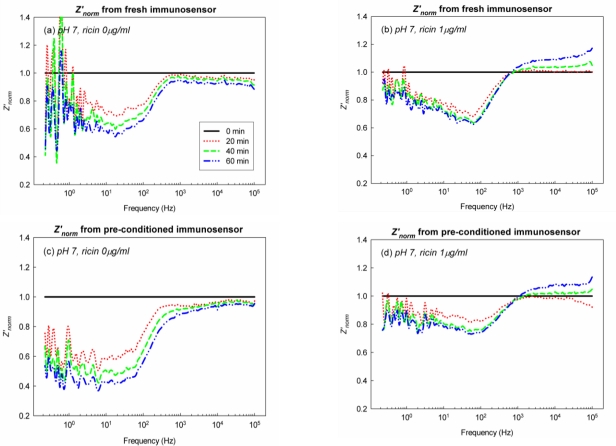
*Z’_norm_* spectra from EIS analysis of EII at pH 7; for the fresh EII with the treatment of **(a)** 0 μg/mL and **(b)** 1 μg/mL of Ag; and for preconditioned-EII with the treatment of **(c)** 0 μg/mL and **(d)** 1 μg/mL of Ag. Ricin, the biological toxin, was used as Ag of EII.

**Figure 5.**
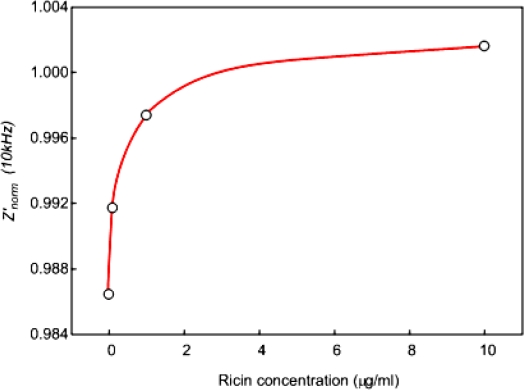
*Z’_norm_* (10 kHz) at 20 mins from EIS analysis of EII in the differently concentrated Ag solutions (0, 0.1, 1, and 10 μg/mL). Ricin, the biological toxin, was used as Ag of EII.

EII was preconditioned in pH 3, 0.3% NaCl solution for 60 mins and washed with pH 7, 0.3% NaCl solution. The immunological activity of immobilized Ab on preconditioned-EII was investigated by the monitoring of immunoreaction in pH 7, Ag 1 μg/mL solution. Figure presents *Z’_norm_* spectra of preconditioned-EII and fresh EII in pH 7, Ag 0 μg/mL and pH 7, Ag 1 μg/mL solutions. *Z’_norm_* spectra were collected every 20 min for 60 mins from EIS analysis under the polarization. Negatively charged ions would be attracted and adsorbed on the surface of EII by the polarization during EIS analysis. By the repetition of EIS analysis, the adsorption and relaxation of negatively charged ions on the surface of EII would occur repeatedly. It was supposed that the hydrophobicity of EII stimulated the relaxation of ions during the recess of EIS analysis. However some hydroxides would not be relaxed but remained on the hydrophobic body of APTES-SAMs through physical adsorption [5–7]. The gradual decrease of *Z’_norm_* in pH 7, Ag 0 μg/mL solution might be due to the adsorption of hydroxides on the surface of EII by the repetition of the polarization (Figure 4(a)). It is certain that the immunoreaction on the surface of EII occurred along with the adsorption of negatively charged ions. *Z’_norm_* of EII in pH 7, Ag 1 μg/mL solution would be affected by the hydroxide adsorption (Figure 4(b)). However the interfacial changes of EII by the immunoreaction between immobilized Ab on EII and Ag in a diagnostic solution overcame the effect of the hydroxide adsorption on *Z’_norm_* thus this resulted in the higher *Z’_norm_* especially from 1 kHz to 100 kHz in pH 7, Ag 1 μg/mL solution than those in pH 7, Ag 0 μg/mL solution (Figure 4(b)). Therefore it was concluded that the hydroxide adsorption on the hydrophobic surface of EII decrease *Z’_norm_* especially from 1 kHz to 100 kHz but the immunoreaction between immobilized Ab on EII and Ag in a diagnostic solution hinders the decrease of *Z’_norm_* by the hydroxide adsorption. Additionally, in order to confirm the effect of the immunoreaction on *Z’_norm_* of EII, *Z’_norm_* at 10 kHz (*Z’_norm_* (10 kHz)) at 20 min of EII in the differently concentrated Ag solutions (0.1–10 μg/mL) were recorded and presented in Figure *Z’_norm_* (10 kHz) at 20 mins of EII was altered dependently on Ag concentration. Accordingly we could confirm that the higher *Z’_norm_* (10 kHz) of EII in pH 7, Ag 1 μg/mL solution than those in pH 7, Ag 0 μg/mL solution was due to the immunoreaction on EII (Figure 4(a,b), and Figure 5), and furthermore EII developed in this study would be valid as the detection method of ricin.

*Z’_norm_* spectra from preconditioned-EII are very similar to those from fresh EII (Figure 4). A gradual decrease of *Z’_norm_* by the hydroxide adsorption was observed from preconditioned-EII in pH 7, Ag 0 μg/mL solution (Figure 4(c)). In addition *Z’_norm_* of the preconditioned-EII from 1 kHz to 100 kHz in pH 7, Ag 1 μg/mL solution was higher than in pH 7, Ag 0 μg/mL solution (Figure 4(d)).

### 3.4. The Effect of Acids on Immunosensing

**Figure 6.**
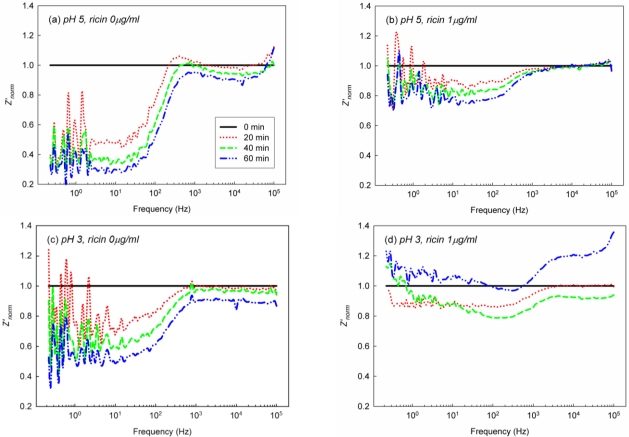
*Z’_norm_* spectra from EIS analysis of EII with the different treatment of Ag concentration; at pH 5, **(a)** 0 μg/mL, **(b)** 1 μg/mL of Ag; and at pH 3, **(c)** 0 μg/mL, **(d)** 1 μg/mL of Ag. Ricin, the biological toxin, was used as Ag of EII.

*Z’_norm_* spectra of EII in acidic environment are described in Figure 6. To investigate the immunoreaction on EII in acid environment, *Z’_norm_* spectra of EII were collected every 20 min for 60 mins in pH 3, pH 5, and pH 7 Ag solutions. The gradual decrease of *Z’_norm_* from 1 kHz to 100 kHz by the hydroxide adsorption was observed in pH 5, Ag 0 μg/mL and pH 3, Ag 0 μg/mL (Figure 6(a,c)). However *Z’_norm_* spectrum of EII was shifted strangely at 60 mins in pH 3, Ag 1 μg/mL (Figure 6(d)) Such a strange shift of *Z’_norm_* spectrum in pH 3, Ag 1 μg/mL would be the influence of acids on immobilized Ab.

It has been discussed that the hydrophobicity of EII would prevent acids from the access to the surface of EII particularly if EII was not under the polarization (Figure 4). However the polarization of EII would make acids at the solid-liquid interface of EII accessible to immobilized Ab on EII. The polarization of EII forced negatively charged ions to be attracted to the aluminum surface of EII through APTES-SAMs (Figure 3). Over the layers of negatively charged ions under the polarization of EII, acids in acidic diagnostic solutions would be diffused and accessed to immobilized Ab, which was cross-linked with APTES-SAMs. Excessive exposure of EII to acidic environment would denaturate immobilized Ab on EII. As denaturated protein tends to adsorb other proteins non-specifically to its structure, the strange shift of *Z’_norm_* spectrum at pH 3, Ag 1 μg/mL would be caused by the non-specific adsorption of Ag on EII, on which immobilized Ab was denaturated by acids (Figure 6(d)).

**Figure 7.**
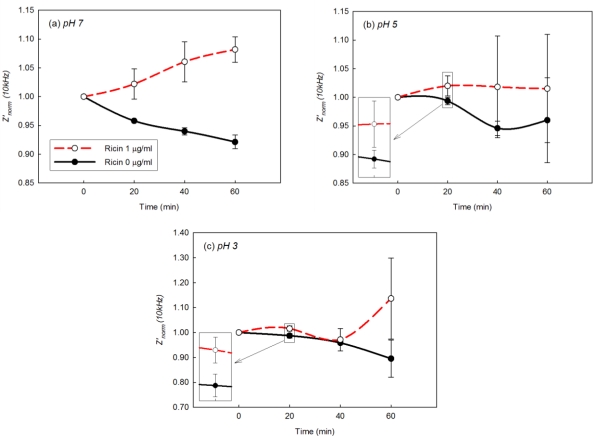
*Z’_norm_* (10 kHz) from EIS analysis of EII in 0 μg/mL and 1 μg/mL of Ag at **(a)** pH 7, **(b)** pH 5, and **(c)** pH 3. Ricin, the biological toxin, was used as the Ag of EII.

Despite of the effect of acids, *Z’_norm_* at 20 mins around 10 kHz in Ag 1 μg/mL solutions at pH 5 and pH 3 were higher than those in Ag 0 μg/mL solutions at pH 5 and pH 3 (Figure 6). Because the difference of *Z’_norm_* at 10 kHz between the presence and the absence of Ag was small, *Z’_norm_* (10 kHz) from the triplicate of EIS of EII at pH 7, pH 5, and pH 3 were re-plotted in Figure 7. As observed from *Z’_norm_* spectra of EII at pH 7 (Figure 4), *Z’_norm_* (10 kHz) was gradually decreased in pH 7, Ag 0 μg/mL solutions as well as *Z’_norm_* (10 kHz) in pH 7, Ag 1 μg/mL solutions was reproducibly higher than those in pH 7, Ag 0 μg/mL solutions (Figure 7(a)). Although *Z’_norm_* (10 kHz) was changed strangely by the effect of acids especially at 40 mins and 60 mins, *Z’_norm_* (10 kHz) at 20 mins in Ag 1 μg/mL solutions at pH 5 and pH 3 were reproducibly higher than those in Ag 0 μg/mL solutions at pH 5 and pH 3 (Figure 7(b,c)). Such higher *Z’_norm_* (10 kHz) at 20 mins would be due to the immunoreaction between immobilized Ab on EII and Ag in diagnostic solutions.

The polarization of EII attracts negatively charged ions and leads acids to access to immobilized Ab as observed in Figure 4 and Figure 6. However, under the polarization, hydroxides also adsorb on hydrophobic body of APTES-SAMs. Since Ab on EII are closely immobilized with APTES-SAMs (Figure 2(c)), hydroxides adsorbed on APTES-SAMs would offset acids, which accessed near immobilized Ab, and let immobilized Ab participate to the immunoreaction with Ag in the acidic environment. But the excessive exposure of immobilized Ab to acids would cause the denaturation of Ab. Therefore hydroxides adsorbed on the hydrophobic surface of EII help to maintain the immunological activity of immobilized Ab in the acidic environment and enable EII to recognize Ag in acidic diagnostic solutions.

**Figure 8.**
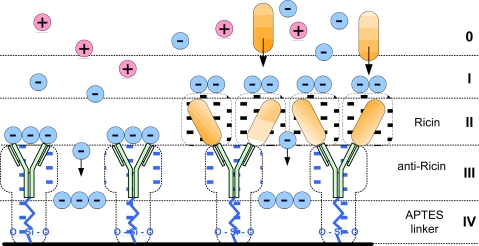
Schematic diagram of anti-Ricin molecules immobilized onto surface and suggested zoned distribution of positive and negative ions from the sample matrix.

Let us consider the transport of solute ions at the sensor’s surface affected by an external electric field. As depicted in Figure 8, we can represent EII sensor’s surface vicinity as a zoned layer comprised from several distinct regions. Starting from the sensor’s surface, the first zone is the surface itself with APTES self-assembling monolayer. The molecular weights of Ab and the blocking agent (gluteraldehyde) are of several orders of magnitude higher than the molecular weight of electric field-driven ions. Therefore, ions (specifically hydroxides) migrating through the protein layer (Ab, Ab/Ag) reach the surface and adsorb to it. This is supported by the finding that the point of zero charge on CV curves corresponds to the isoelectric point of pure aluminum [15]. The next layer is the layer of immobilized Ab (anti-Ricin). In terms of ionic transport, interaction of migrating ions with this layer can be interpreted as additional mass-transfer resistance. The mechanism of this interaction is two-fold: first, decreasing of ionic mobility due to changes in medium viscosity, and second, electrostatic interaction of ions with protein groups and their hydrated shells. Anti-Ricin protein molecules are formed from a mixture of polar and non-polar molecular groups. Water molecules surrounding them become ordered, creating hydration shells around proteins with high proton transfer rates. Water in these shells is 10%–20% denser than the bulk water [16]. Furthermore, the internal molecular motion in proteins, crucial for the immunoreaction to occur, strongly depends on the hydration process.

An external (faced outwards) part of Ab layer is also subject to the adsorption process. Therefore, overall transport of charged particles at the electrode surface without the immunoreaction consists of electromigration of ions through Ab layer and their adsorption onto protein layer and metal substrate. Characteristic shapes of CV curves corresponding to this mechanism have been observed in our experiments (see Figure 3).

Presence of Ag in the solution results in the formation of Ab/Ag complexes that can be interpreted as formation of additional Ag layer over the Ab layer. The properties of this new layer significantly differ from the Ab layer beneath. As reported in the literature, isoelectric point, *i.e.*, surface charge of Ricin differs from that of anti-Ricin. This difference has been used to electrophoretically separate anti-gene from Ab/Ag complexes [17]. Therefore, the value of electrodiffusion resistance of Ag layer differs from that of Ab layer. Additional factor, which could significantly impact the distribution of ions near the sensor’s surface and their transport, is that the molecular weight of Ricin is much smaller than that of corresponding Ab. Therefore, charge distribution and charge density over Ag layer significantly differ from those of Ab layer, which results in local changes of the pH making immunoreaction possible. The distribution of ions shown in this diagram also reflects fact that both Ricin and anti-Ricin molecules are positively charged with isoelectric point around pH 7.2 [18].

### 3.5. Application of EII to Acidic Foods

**Figure 9.**
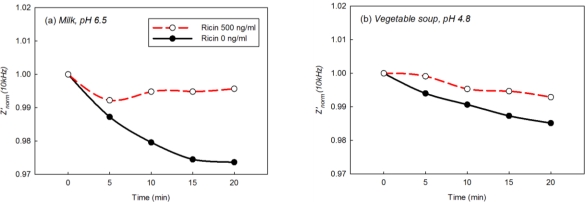

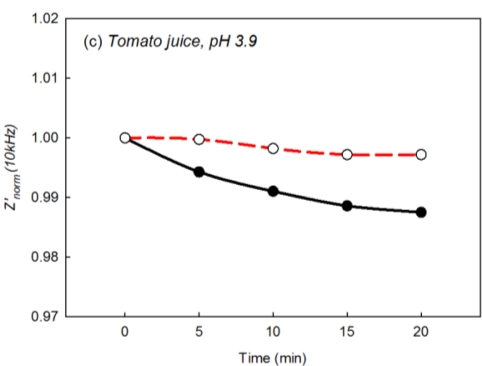
*Z’_norm_* (10 kHz) from EIS analysis of EII in **(a)** milk, **(b)** vegetable soup, and **(c)** tomato juice containing 0 ng/mL or 500 ng/mL of Ag. Ricin, the biological toxin, was used as the Ag of EII.

Immunosensing performance of EII was investigated in acidic foods at different pH: milk (pH 6.5), vegetable soup (pH 4.8), and tomato juice (pH 3.9), which can be categorized to neutral, low acid, and acid foods [3,4]. *Z’_norm_* (10 kHz) of EII in acidic foods was monitored every 5 min for 20 mins (Figure 9). While the values of *Z’_norm_* (10 kHz) by immunoreaction was gradually increased in pH 7, 0.3% NaCl solution with Ag 1 μg/mL in Figure 7(a), those in milk in Figure 9(a), didn’t show the similar increasing trend due to the effect of complex food matrix on impedimetric behaviors of EII. However *Z’_norm_* (10 kHz) at the presence of Ag in acidic foods were higher than those at the absence of Ag (Figure 9). As the higher *Z’_norm_* (10 kHz) is regarded to be caused by the immunoreaction between immobilized Ab on EII and Ag in acidic foods, we conclude that EII, of which the surface is treated hydrophobically, is applicable for the direct detection of Ag in acidic foods without pH adjustment.

## 4. Conclusion

As the hydrophobically modified EII maintained its immunological activity in the acidic environment, EII was capable of directly detecting the biological toxin without pH adjustment. The changes in the physical and/or chemical properties at the solid-liquid interface of EII by the immunoreaction between immobilized Ab on EII and Ag in a diagnostic solution responded specifically to *Z’* under the polarization of EII *versus* reference electrode. The values of *Z’_norm_* (10 kHz) from EII at the presence of the biological toxin were reproducibly higher than those at the absence of toxin. Under the polarization of EII, acids and hydroxides showed different behaviors to the hydrophobic surface of EII. Hydroxides, which were adsorbed at the water-hydrophobic interface of EII by the polarization, would offset the effect of acids on the immunological activity of immobilized Ab on EII, and this allows immobilized Ab to participate in the immunoreaction in acidic environment. In addition, the hydrophobically modified EII was potentially applicable to the detection of the biological toxin in acidic foods as rapidly as 20 mins.
